# MediBoost: a Patient Stratification Tool for Interpretable Decision Making in the Era of Precision Medicine

**DOI:** 10.1038/srep37854

**Published:** 2016-11-30

**Authors:** Gilmer Valdes, José Marcio Luna, Eric Eaton, Charles B. Simone, Lyle H. Ungar, Timothy D. Solberg

**Affiliations:** 1 Radiation Oncology Department, University of California, San Francisco, CA, 94115, USA; 2 Department of Radiation Oncology, Perelman Center for Advance Medicine, University of Pennsylvania, Philadelphia, PA, 19104, USA; 3 Department of Computer and Information Science, University of Pennsylvania, Philadelphia, PA, 19104, USA

## Abstract

Machine learning algorithms that are both interpretable and accurate are essential in applications such as medicine where errors can have a dire consequence. Unfortunately, there is currently a tradeoff between accuracy and interpretability among state-of-the-art methods. Decision trees are interpretable and are therefore used extensively throughout medicine for stratifying patients. Current decision tree algorithms, however, are consistently outperformed in accuracy by other, less-interpretable machine learning models, such as ensemble methods. We present MediBoost, a novel framework for constructing decision trees that retain interpretability while having accuracy similar to ensemble methods, and compare MediBoost’s performance to that of conventional decision trees and ensemble methods on 13 medical classification problems. MediBoost significantly outperformed current decision tree algorithms in 11 out of 13 problems, giving accuracy comparable to ensemble methods. The resulting trees are of the same type as decision trees used throughout clinical practice but have the advantage of improved accuracy. Our algorithm thus gives the best of both worlds: it grows a single, highly interpretable tree that has the high accuracy of ensemble methods.

The *stratification* of patients into subpopulations is at the core of clinical decision-making and clinical trial design in medicine^1^,^2^,^3^. With the increased focus on precision medicine, the stratification of patients into subpopulations is essential for increased diagnostic and treatment efficacy, including targeted gene therapies, diverse disease presentations, and accurate prognosis. Better patient stratification is also needed to improve the unacceptably low success rates of some clinical trials^1^,^2^,^4^. If clinical trials are performed in a poorly stratified cohort of patients, effective targeted therapies will only be discovered when the incidence of the responsive subpopulation and the effect size within this group is sufficiently high^4^. This scenario increases the size of clinical trials to unaffordable levels and currently results in frequent failure.

Patient stratification involves the integration of complex data structures that include gene-expression patterns, individual proteins, proteomics patterns, metabolomics, histology or imaging^2^, all of which machine learning algorithms can correctly analyze. Other sources of information, however, such as those from electronic medical records, scientific literature, and physician experience and intuition, are more difficult to integrate. For this reason, *interpretability* is a core requirement for machine learned models used in medicine. Moreover, all such learned models have some degree of inaccuracy, which leaves healthcare providers with the question of what to do when their intuition and experience disagree with the prediction of a model. Most human experts will override the model in these cases, since misclassification in medicine can have adverse consequences. In fact, the most widely used medical scoring and classification systems are highly interpretable but are not optimized for accuracy^5^,^6^,^7^,^8^. Both patients and physicians need to understand the reasons behind a prediction, in order to take an appropriate course of treatment that goes beyond predicted outcome and incorporates the expectation of patients^3^.

The requirements of stratification and interpretability are the reason why decision trees produced by machine learning algorithms such as C4.5, ID3, and CART, are so widely used in medicine^9^,^10^,^11^,^12^,^13^,^14^,^15^,^16^. Decision trees simulate the way physicians think by stratifying a patient population into subpopulations based on few conditional statements (*i.e.,* if-then rules) about the patient^5^,^6^,^7^,^8^,^9^,^10^,^11^,^12^,^13^,^14^,^15^,^16^. In a decision tree, these rules are represented by nodes organized in a tree-based structure, leading to a prediction (Fig. 1). The interpretability of decision trees allows physicians to understand why a prediction or stratification is being made, providing an account of the reasons behind the decision to subsequently accept or override the model’s output. This interaction between humans and algorithms can provide more accurate and reliable diagnostics and personalized therapeutics, and greatly improve clinical trial design, as compared with either method alone. The historical challenge to machine learning applications, however, is the *tradeoff* between accuracy and interpretability^3^,^17^,^18^,^19^. Decision trees are consistently outperformed by ensemble learning methods, such as AdaBoost, gradient boosting, and random forests^20^,^21^,^22^,^23^, which combine multiple classifiers into a highly accurate but less interpretable model. In this more complex models, interpretability is sought by assigning unbiased estimation of the variable importance^20^,^21^,^22^,^23^. Within the medical community, however, a classifier is considered to be interpretable if one can explain its classification by a conjunction of conditional statements, *i.e.,* if-then rules, about the collected data, in our case, data used for patient stratification. Under this definition, standard decision trees, such as those learned by ID3 or CART, are considered interpretable but ensemble methods are not.

In this article, we present a framework for constructing decision trees that have equivalent accuracy to ensemble methods while maintaining high interpretability. This unique combination of model accuracy and interpretability addresses a long-standing challenge in machine learning that is essential for medical applications. This framework is referred to as *MediBoost* for its application to medicine. The resulting trees can directly replace the existing decision trees used throughout clinical practice, significantly increasing their accuracy while providing equivalent interpretability. Additionally, the applications of our algorithm are not limited to the medical field; it could be used in any other application that employs decision trees.

## Methods

### MediBoost Framework

MediBoost is new framework to build accurate decision trees based on boosting^21^,^22^,^23^. We first discuss a classic boosting method, the AdaBoost algorithm^21^, and then show how boosting can be used to derive the MediBoost framework. AdaBoost combines *weak learners*, which are classifiers whose prediction is only required to be slightly better than random guessing, via a weighted sum to produce a strong classifier^21^. AdaBoost takes as input a set of labeled data and iteratively trains a set of *T* decision stumps (single node decision trees) as the weak learners {*h*_1, …,_*h*_T_} in a stage-wise approach, where each subsequent learner favors correct classification of those data instances that are misclassified by previous learners. Each decision stump *h*_*t*_ splits the data via a predicate *a*_*t*_ that focuses on a particular attribute of each data instance ***x*** (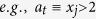), yielding a prediction 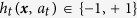. Given a new data instance characterized by an observation vector ***x***, AdaBoost predicts the class label 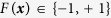 for that instance as:


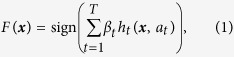


where the weight 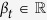 of each decision stump *h*_*t*_ depends upon its weighted classification error on the training data^21^. Decision stumps are ideal weak learners due to their ability to incorporate categorical or continuous variables and missing data, because they are robust to outliers, and because they perform internal feature selection^24^.

The crucial idea behind MediBoost is simple: an ensemble of decision stumps (one-node decision trees), such as that produced by AdaBoost, can be rewritten as a decision tree by considering all possible combinations of predictions made by each ensemble member (Fig. S1). MediBoost builds an interpretable tree, rather than a weighted sum of many weak learners, by constructing a tree where each path from the root to a terminal node contains *T* nodes and represents a particular combination of the prediction of the ensemble members. The tree is constructed by recursively adding branches such that at each branch, from the root to a terminal node, the stumps *h*_*1*, …,_*h*_*T*_ from the AdaBoost ensemble are assigned (Fig. S1), pairing each node of the decision tree with a particular attribute of the data and corresponding threshold. The final classification at each terminal node is then given by Equation 1. See Algorithms I and II in the Supplementary Materials for details. The resulting tree has depth T, and hence 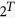 branches. In practice, these trees can be severely pruned; all branches that do not change the sign of the classification of its parent nodes can be pruned without loss of accuracy. Because MediBoost at its core is a boosting framework, different boosting methods including gradient boosting, and additive logistic regression with different loss functions^21^,^22^,^23^ can be used to construct specific MediBoost decision tree induction algorithms. In the Supplementary Materials, we include the derivation of the general Mediboost algorithm, Gradient Mediboost (GMB), as well as two specific MediBoost algorithms: (1) MediAdaBoost (MAB) using additive logistic regression and (2) LikelihoodMediBoost (LMB) using gradient boosting. MAB is attractive due to its simplicity and similarity to the original boosting algorithm, AdaBoost, whereas LMB is expected to result in trees that are more accurate than MAB. Similar to AdaBoost, MAB, can be obtained by minimizing an exponential loss function using additive logistic regression^22^ with the addition of a membership function that describes the degree of belonging of a certain observation to a given node. MAB thus, finds each node of the decision tree by focusing on instances with higher probability of belonging to that node, as in fuzzy logic^25^, rather than only on the data instances that previous nodes have misclassified, as in AdaBoost^21^. LMB is obtained using gradient boosting^23^ by finding the split that minimizes the quadratic error of the first derivative of the binomial log-likelihood loss function and determining the coefficients according to the same framework.

Reinterpreting MediBoost using gradient boosting not only allows different loss functions, but provides the necessary mechanisms to add regularization beyond penalizing for the size of the tree (as is sometimes done in regular decision trees^10^,^24^) in order to obtain better generalization accuracy. A detailed mathematical derivation of these algorithms and their pseudocodes are included in the Supplementary Materials.

Implementations of the MAB and LMB algorithms are available at www.mediboostml.com.

### Experiments

The MAB and LMB MediBoost algorithms were compared to standard decision tree induction (ID3, CART) and ensemble methods (LogitBoost and Random Forests) on 13 data sets, corresponding to all binary classification problems in the field of Life Sciences within the UCI Repository (http://archive.ics.uci.edu/ml/ - Table S1). For each data set, any missing values were imputed with either the mean or the mode of the corresponding feature, depending on whether the features were continuous or categorical. We added additional binary features, one per each original feature, to encode whether or not the corresponding value was missing. Results were averaged over 5 trials of 5-fold cross-validation on each data set, recording the balanced cross validation error on the held-out test fold. Additionally, the area under the curve (AUC) was also determined in a similar fashion for each algorithm. Moreover, a permutation test was performed were the labels were randomly muted 100 times and the probability of obtaining a better AUC calculated. Each algorithm has a number of hyperparameters, which were tuned using an additional 5-fold cross-validation on the training data in each case. Therefore, the model was constructed using all available training folds and evaluated on the test fold. The hyperparameters adjusted for each algorithm are:

- **MediBoost (MAD and LMB):** tree depth and acceleration parameter.
- **ID3:** tree depth.
- **CART:** tree depth.
- **LogitBoost:** Number of stump trees on the ensemble.
- **Random Forests:** Number of variables selected in each random sub-sampling.

In addition, LogitBoost used decision stumps as the weak learners with a learning rate of 0.1, and Random Forests used 300 decision trees in the ensemble. The MediBoost algorithms were run with learning rates of 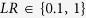 and λ = 0.

## Results

The performance of LMB and MAB were compared with CART, LogitBoost, Random Forests as implemented in Matlab**®** R2015a, and our own implementation of ID3. All results were averaged over 5-fold cross-validation on the data sets, with hyper-parameters chosen in an additional 5-fold cross-validation on the training folds as explained in the Methods section.

As shown in Table 1, LMB, with its default settings, performs better than its decision tree cousins (ID3 and CART) when the balanced classification error is compared in 11 out of the 13 medical problems. If the AUC is compared, then MediBoost performs better than current decision tree algorithms in 12 out of 13 problems, Table 2. A graphical comparison of the balanced cross-validation error values and AUC is also shown in Fig. 2 and Fig. 3. These results are statistically significant in a two-way sign-to-sign test^26^,^27^. In one of the problems where the default LMB was not superior, the standard decision trees also outperformed the ensemble methods. In a three-way ANOVA comparison of the balanced cross-validation errors between LMB, ID3 and CART across all problems, LMB was significantly better than ID3 (p = 10^−8^) and CART (p = 0.014). In comparison to the ensemble methods, LMB was indistinguishable from LogitBoost (p = 0.44) and worse than random forests (p = 0.0004). In a three-way Friedman test^28^, more robust than ANOVA when comparing algorithms, LMB was significantly better than ID3 (p = 0.006) and CART (p = 0.09) at the 90% confidence interval, but not significantly different from either LogitBoost (p = 0.97) or random forests (p = 0.30). Similar results were obtained when LMB was run with a learning rate of 0.1 (Fig. S2). Additionally, MAB gave slightly but not statistically significantly poorer results to those obtained using LMB (Table 1 and Fig. S3.). If the AUC are compared using the Wilcoxon sign rank test with the Bonferroni adjustment for multiple comparison, then MediBoost is significantly better than ID3 (p = 8.69 × 10^−10^) and CART (p = 8.89 × 10^−9^) but not significantly different from LogitBoost (p = 0.85). Random forests was indeed significantly better than MediBoost (p = 1.5 × 10^−6^) when AUC were compared and the clear winner.

Further, MediBoost retains the interpretability of regular decision trees (Fig. 4). This interpretability is not only the result of it producing a tree-based model, but also in the significant shrinkage obtained compared to boosting. This shrinkage is due to the introduction of the membership function controlled by an acceleration parameter, elimination of impossible paths during the learning process, and a post-training pruning approach that does not change the accuracy of the model (as described in the Supplementary Materials). Once a deep MediBoost tree is grown (*e.g.,* with a depth of 15 nodes at each branch), all branches that do not change the sign of the classification of its parent nodes can be pruned without loss of accuracy. This pruning approach has been used to represent the MediBoost tree in Fig. 4.

Additionally, the effect of varying the acceleration parameter for both LMB and MAB in different data sets was evaluated (Fig. 5). In all cases, our results show that the training errors decrease as the acceleration parameter increases, while the test error remains the same or decreases. These results demonstrate that the performance of the resulting MediBoost tree is relatively insensitive to small changes in the acceleration parameter, allowing it to be effectively tuned to reduce the size of the tree for better interpretability with a minimal impact on accuracy. Finally, in order to show MediBoost robustness a permutation test was performed where labels were randomly permuted 100 times and the probability of obtaining a better AUC than in the original analysis calculated for all algorithms together with the mean value and standard deviation of the permuted AUC. This data in shown on Table S6. As it can be observed all algorithms show similar robustness. The estimated probability of obtaining an AUC in the random permutation experiment bigger than the obtained through the analysis of the data using MediBoost was < 0.01 for all data sets except for the Fertility dataset when this value was 0.1.

## Discussion

**Figure 1:**
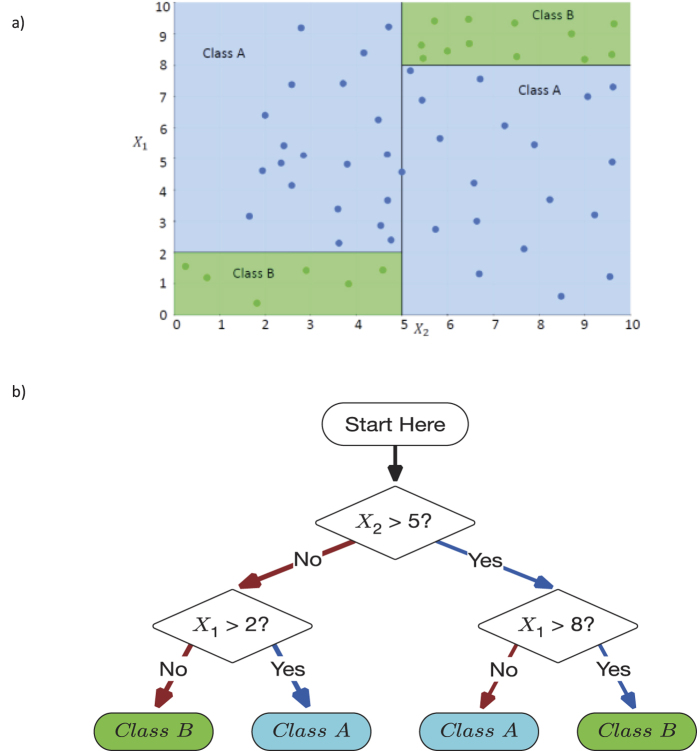
An example decision tree on a toy data set, showing (**a**) the induced decision surface (shaded regions) and the set of 2D training data, where the color of each data instance represents its class label, and (**b**) the corresponding decision tree, composed of three decision nodes to partition the data into the four subregions.

**Figure 2:**
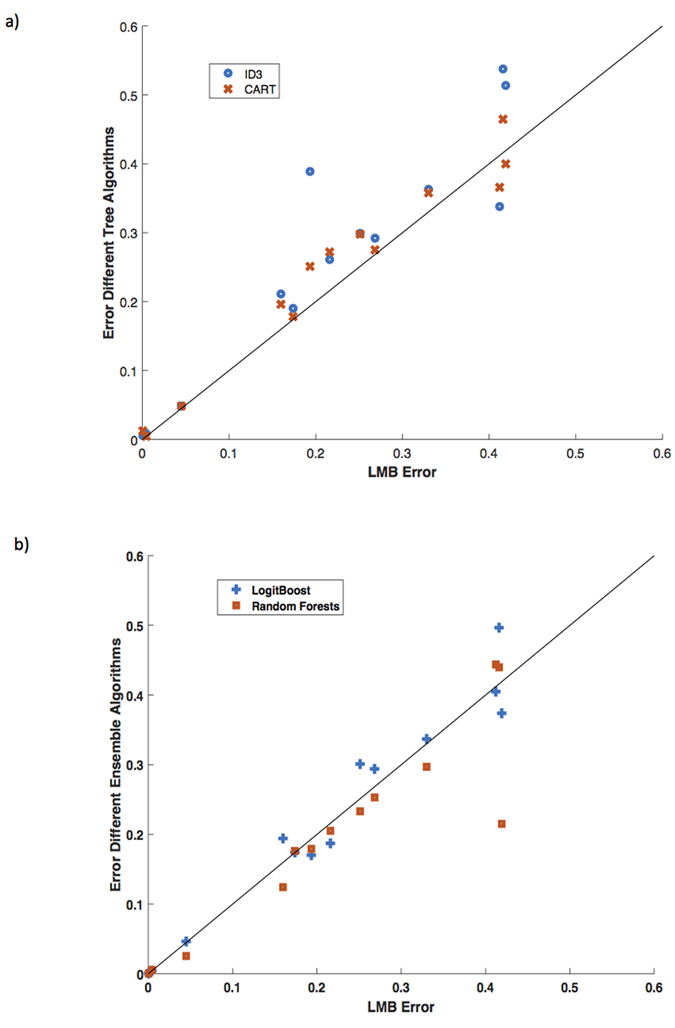
Comparison of LMB using balanced classification error vs (**a**) different tree algorithms (ID3 and CART) and (**b**) different ensemble methods (LogitBoost and Random Forests) on 13 medical datasets. Points above the black line indicate results where LMB was better. LMB is significantly better than the decision tree algorithms and indistinguishable from ensemble methods.

**Figure 3:**
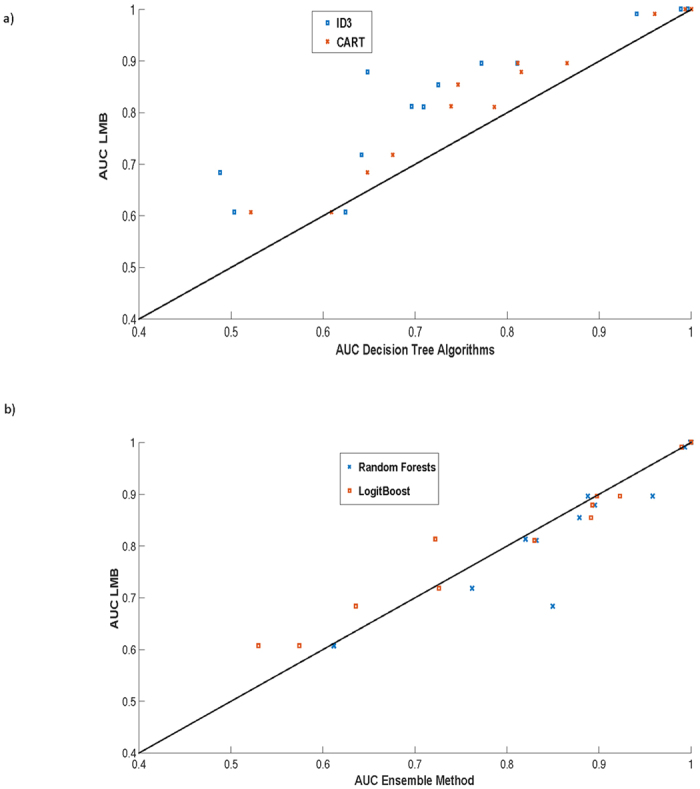
Comparison of LMB using AUC error vs (**a**) different tree algorithms (ID3 and CART) and (**b**) different ensemble methods (LogitBoost and Random Forests) on 13 medical datasets. Points above the black line indicate results where LMB was better.

**Figure 4:**
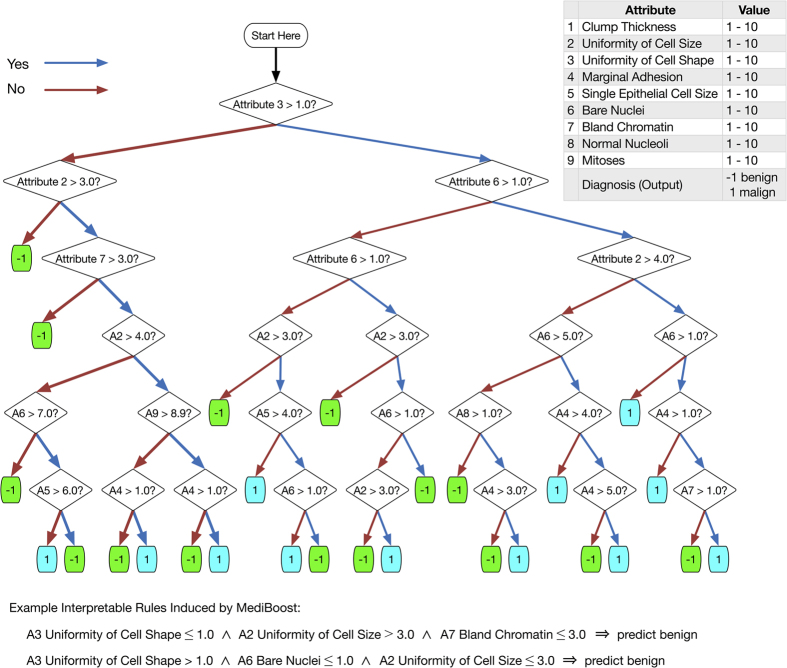
MediBoost decision tree obtained using LMB on the Wisconsin Breast Cancer data set after pruning. “Attribute” has been changed to “A” in deeper nodes for simplicity.

**Figure 5:**
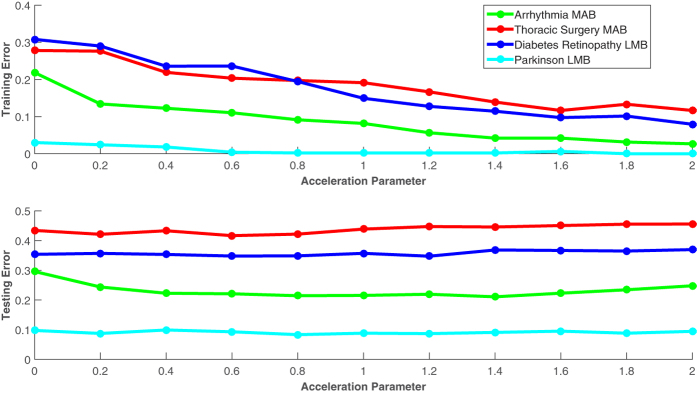
Effect of acceleration parameter on the training and testing error for four different data sets and two different MediBoost algorithms (MAB and LMB). In all cases, the training error decreases as the acceleration parameter increases (accelerating the convergence of the algorithm) while the testing error improves or remains the same.

Traditional decision trees perform recursive partitioning in order to arrive at a prediction. At each node of the tree, the observed data are further subdivided so that as one goes farther down the tree, each branch has fewer and fewer observations, as illustrated in Fig. 1. This strongly limits the possible depth of the tree as the number of available observations typically shrinks exponentially with tree depth. In this ‘greedy search’ over data partitions, assigning an observation on the first few nodes of the tree to incorrect branches can greatly reduce the accuracy of the resulting model^24^. MediBoost trees are constructed using a different mathematical framework, called boosting, in which each node focuses on observations that previous nodes have not separated correctly^21^,^22^,^23^. Additionally, in order to obtain a smaller tree, which is a key issue in maintaining interpretability, MediBoost penalizes the weights of observations assigned to different branches through the novel introduction of a membership function, forming a relative “soft” recursive partition similar to decision trees grown using fuzzy logic^25^. In MediBoost, no hard partitioning is performed, and all observations contribute to all decision nodes. The specialized reader will identify that each path through a MediBoost tree represents a different ensemble, similar to those generated by AdaBoost or gradient boosting, as illustrated in Fig. S1^21^,^22^,^23^. This is fundamentally different from previous decision tree learning algorithms^29^ and is the primary reason for the improved accuracy of MediBoost with respect to current decision tree algorithms. We conclude that MediBoost in its various forms is significantly better than standard decision tree induction algorithms and has comparable accuracy to ensemble methods, based on the two way sign-to-sign, three-way ANOVA, Friedman and Wilcoxon tests shown above. In two of the statistical tests for both balanced cross-validation error and AUC, our decision tree algorithm, in its current form was inferior to random forests. This is consistent with the observations of Caruana *et al*.^30^, who showed that boosted stumps, the structure currently used in MediBoost nodes, are inferior to random forests. Although we present MediBoost algorithms with only two branches per node in this paper (stumps) for simplicity, it could easily be extended to multi-branch nodes, where each node will represent a tree similar to those used in boosted trees with the corresponding improvement in accuracy as shown by Caruana *et al*.^30^. When only stumps are used, MediBoost only takes into account additive effects but random forests is taking into account both additive and interaction effects. If multi-branch nodes are used, however, interaction effects will be taken in to account by MediBoost. In this case, it is expected that MediBoost will be on average equally or more accurate than random forests^30^. Additionally, the magnitude of the difference was bigger than 0.03 in only 4 problems out of 13 which might indicate that MediBoost might still be the prefer option in most cases, Table S5.

Moreover, MediBoost has been generalized to any loss functions, it can also be easily extended to regression, multi-class or survival analysis. This is one of the advantages over other methods like Bayesian Rule Lists, though MediBoost rules could be larger and more complex in this case^31^. Finally, healthcare providers, patients, and biomedical researchers should not be discouraged by the mathematical complexity of the underlying our method-while the mathematical framework of MediBoost is complex, its output, a single tree for any given problem, can be understood with little mathematical knowledge. In fact, MediBoost produces decision trees that can immediately replace those used in current clinical practice/research, a sub-sample of which are referenced in this paper. If MAB and LMB are applied to these previously published medical problems, we predict that more accurate decision trees will be obtained in the majority of problems, with a corresponding positive impact on clinical practice/research. MediBoost thus gives the best of both worlds: it grows a single, highly interpretable tree that has the high accuracy of ensemble methods.

## Conclusion

MediBoost results in trees that perform highly interpretable patient stratification while obtaining excellent accuracy that is similar to ensemble methods. In the era of precision medicine, MediBoost can empower doctors, patients, and researchers alike to make accurate and interpretable data-driven clinical decisions, and to improve the design and success rates of clinical trials.

## Additional Information

**How to cite this article**: Valdes, G. *et al*. MediBoost: a Patient Stratification Tool for Interpretable Decision Making in the Era of Precision Medicine. *Sci. Rep.* **6**, 37854; doi: 10.1038/srep37854 (2016).

**Publisher's note:** Springer Nature remains neutral with regard to jurisdictional claims in published maps and institutional affiliations.

## Supplementary Material

Supplementary Information

## Figures and Tables

**Table 1:** Comparing algorithms using the balanced cross-validation error.

LMB vs	ID3	CART	LogitBoost	Random Forests
wins	12	11	7	4
losses	1	2	4	8
ties	0	0	2	1
**MAB vs**	**ID3**	**CART**	**LogitBoost**	**Random Forests**
wins	11	10	5	4
losses	1	2	6	8
ties	1	1	2	1

Results of LMB and MAB MediBoost algorithms vs different decision tree (ID3 & CART) and ensemble learning (LogitBoost & Random Forests) algorithms on 13 medical data sets. showing the number of data sets where the MediBoost had better, worse, or equivalent accuracy.

**Table 2:** Comparing algorithms using the AUC.

LMB vs	ID3	CART	LogitBoost	Random Forests
wins	12	11	5	1
losses	1	1	6	10
ties	0	1	2	2

Results of LMB vs different decision tree (ID3 & CART) and ensemble learning (LogitBoost & Random Forests) algorithms on 13 medical data sets, showing the number of data sets where the MediBoost had better, worse, or equivalent accuracy.
