# Supplementary material for: MediBoost: a Patient Stratification Tool for Interpretable Decision Making in the Era of Precision Medicine
Source: Sci Rep. 2016 Nov 30;6:37854. doi: 10.1038/srep37854 (PMC5129017; doi:10.1038/srep37854)
Supplement: Supplementary Information [file srep37854-s1.pdf]

*To whom correspondence should be addressed; Email: gilmer.valdes@ucsf.edu.

Supplementary Material for

MediBoost: a Patient Stratification Tool for Interpretable Decision Making in the Era of Precision Medicine

Gilmer Valdes*, José Marcio Luna, Eric Eaton, Charles B. Simone II,
Lyle H. Ungar, Timothy D. Solberg

*Corresponding author. E-mail: gilmer.valdes@ucsf.edu

This PDF file includes:

- Supplementary Text
- Table S1-S4
- Figures S1-S3

Implementations of two MediBoost algorithms are available at:
https://www.mediboostml.com

MediBoost: a Patient Stratification Tool for Interpretable Decision Making in the Era of Precision Medicine

Supplemental Material

In this Supplemental Material, we first describe the representation of AdaBoost with decision stumps as a decision tree, and then derive the MediBoost framework based on this representation in combination with gradient boosting and fuzzy logic. We provide details on two MediBoost algorithms: *MediAdaBoost* (MAB), which is based on AdaBoost, and *LikelihoodMediBoost* (LMB), which is based on boosting with the log-binomial likelihood loss function. We also show how the MediBoost framework can easily incorporate regularization and shrinkage. Finally, we describe our experimental methodology and present additional results comparing MediBoost to standard decision tree induction and boosted ensembles.

S1 Introduction

In this supplement, we derive the MediBoost framework for growing interpretable decision trees via boosting. The resulting MediBoost trees obtain comparable accuracy to current ensemble methods, such as AdaBoost and Random Forests, while maintaining the interpretability so necessary for their application to fields like clinical medicine.

We focus on a classification setting, in which we are given a set of training data $\boldsymbol{X} = \{\boldsymbol{x}_1, \ldots, \boldsymbol{x}_N\}$ with corresponding binary labels $\boldsymbol{y} = \{y_1, \ldots, y_N\}$ such that $y_i \in \{-1, +1\}$. Each instance $\boldsymbol{x}_i \in \boldsymbol{X}$ lies in some d-dimensional feature space $\mathcal{X}$, which may include a mix of real-valued, discrete, and categorical attributes. We assume that the labels are given according to some "true" function $F^* : \mathcal{X} \mapsto \{-1, +1\}$, and our goal is to obtain an approximation F of that true function from the labeled training data under some loss function $L(y, F(\boldsymbol{x}))$.

In addition, we use a notion of interpretability that is common in the medical community, considering a classifier to be *interpretable* if we can explain its classification by a conjunction of a few simple questions about the data. Under this definition, standard decision trees (such

as those learned by ID3 or CART) are considered interpretable. In contrast, typical boosting methods and Random Forests produce an unstructured set of weighted hypotheses that can obfuscate correlations among the features, sacrificing interpretability for improved predictive performance. We show that MediBoost trees are interpretable, while obtaining predictive performance comparable to ensemble methods.

S2 Representation of AdaBoost as a Decision Tree

As a precursor to the MediBoost framework, we first consider a method for representing an AdaBoost ensemble classifier with decision stumps as an interpretable decision tree. However, this naive representation has substantial computational and space complexities that are reduced in the MediBoost algorithms described in later sections.

AdaBoost (9) iteratively trains a set of T weak learners $\{h_1, \ldots, h_T\}$ to yield an ensemble classifier $F(\boldsymbol{x})$ that predicts the class label for an observed instance $\boldsymbol{x}$ as:

$$F(\boldsymbol{x}) = \operatorname{sign}\left(\sum_{t=1}^{T} \beta_t h_t(\boldsymbol{x}, a_t) \right) \quad , \tag{1}$$

where predicate a_t represents a threshold or equivalence test on a particular feature of the vector $\boldsymbol{x}$ (e.g., $a_t \equiv$ "$x_j > 3.4$"), and each ensemble member contributes a prediction $h_t(\boldsymbol{x}, a_t) \in \{-1, +1\}$ with a weight of $\beta_t \in \mathbb{R}$ that depends upon its training error. Since each h_t outputs a binary prediction, we can rewrite the model learned by AdaBoost as a complete binary tree with height T by assigning h_t to all internal nodes at depth $t-1$ with a corresponding weight of β_t. The decision at each internal node is given by $h_t(\boldsymbol{x}, a_t)$, and the prediction at each terminal node is given by $F(\boldsymbol{x})$ — the characteristic equation of AdaBoost (9). Essentially, each path from the root to a terminal node represents the same ensemble, but tracking the unique combination of predictions made by each h_t. The resulting tree is illustrated in Fig. S1.

The MediBoost framework is based upon this decision tree representation of a boosted ensemble of decision stumps, and enables us to adapt different boosting algorithms to create different MediBoost algorithms. The trivial representation of AdaBoost as a tree, however, likely results in trees that are accurate but too large to be interpretable. MediBoost remedies this issue by 1.) introducing diversity into the ensemble represented by each path through the tree via a membership function that accelerates convergence to a decision, and 2.) pruning the tree in a manner that does not affect the tree's predictions, as explained below.

S3 MediAdaBoost (MAB)

The MediBoost framework merges the concepts of decision trees, boosting, and fuzzy logic by growing decision trees using boosting with the addition of an acceleration term based on

Algorithm 1 MediAdaBoost($\boldsymbol{X}, \boldsymbol{y}, \boldsymbol{w}_t, \boldsymbol{\mathfrak{D}}, t, T, F_t$)

Inputs:

- training data $\boldsymbol{X} = \{\boldsymbol{x}_1, \ldots, \boldsymbol{x}_n\}$ with corresponding labels $\boldsymbol{y} = \{y_1, \ldots, y_n\}$, where $\boldsymbol{x}_i \in \mathcal{X}$ for some d-dimensional feature space $\mathcal{X}$, and $y_i \in \{-1, +1\}$
- instance weights $\boldsymbol{w}_t \in \mathbb{R}^n$, which defaults to a uniform distribution initially
- valid domain of each attribute $\boldsymbol{\mathfrak{D}} = \{\mathfrak{D}_1, \ldots, \mathfrak{D}_d\}$ (for continuous attributes, $\mathfrak{D}_i$ is a valid interval; for discrete or categorical attributes, $\mathfrak{D}_i$ is a set of valid values)
- node index t, which defaults to 1
- total number of boosting iterations T
- accumulated weighted sum of predictions F_t, which defaults to 0 initially

Outputs: the root node of a decision tree

1: Create a root node N_t for the subtree

2: If $t > T$, Return the single-node subtree N_t with label sign(F_t)

3: Fit weak classifier $h_t(\boldsymbol{x}, a_t) : \mathcal{X} \mapsto \{-1, +1\}$ by finding the stump with decision rule a_t on attribute $attr$ that minimizes the weighted least square error on $(\boldsymbol{X}, \boldsymbol{y})$ with weights $\boldsymbol{w}_t$

4: Calculate the error of the node as $err_t \leftarrow \sum_i w_t(i) \mathbb{1}(y_i \neq h_t(\boldsymbol{x}_i, a_t))$

5: Set $\beta_t \leftarrow \frac{1}{2} \log \left(\frac{1 - err_t}{err_t} \right)$

6: Update the instance weights for the left (-) and right (+) subtrees:

   a. $w_{t+1}^{-}(i) \leftarrow \frac{1}{Z_t^{-}} w_t(i) \exp\left(-\beta_t y_i h_t(\boldsymbol{x}_i, a_t) - A \mathbb{1}(h_t(\boldsymbol{x}_i, a_t) = +1)\right) \ \forall \boldsymbol{x}_i \in \boldsymbol{X}$

   b. $w_{t+1}^{+}(i) \leftarrow \frac{1}{Z_t^{+}} w_t(i) \exp\left(-\beta_t y_i h_t(\boldsymbol{x}_i, a_t) - A \mathbb{1}(h_t(\boldsymbol{x}_i, a_t) = -1)\right) \ \forall \boldsymbol{x}_i \in \boldsymbol{X}$,

   where Z_t^{-} and Z_t^{+} normalize $\boldsymbol{w}_{t+1}^{-}$ and $\boldsymbol{w}_{t+1}^{+}$ respectively to be distributions, and the acceleration constant A penalizes instances where $h_t(\boldsymbol{x}, a_t)$ disagrees with the branch.

7: Let $\mathfrak{D}(a_t)$ represent the domain of attribute $attr$ when a_t is true, and $\mathfrak{D}^c(a_t)$ its complement

8: Compute valid domains for all attributes for the left (-) and right (+) subtrees:

   a. Let $\mathfrak{D}_{attr}^{-} \leftarrow \mathfrak{D}_{attr} \bigcap \mathfrak{D}^c(a_t)$ and $\boldsymbol{\mathfrak{D}}^{-} \leftarrow \left\{\mathfrak{D}_1, \ldots, \mathfrak{D}_{attr-1}, \mathfrak{D}_{attr}^{-}, \mathfrak{D}_{attr+1}, \ldots, \mathfrak{D}_d\right\}$

   b. Let $\mathfrak{D}_{attr}^{+} \leftarrow \mathfrak{D}_{attr} \bigcap \mathfrak{D}(a_t)$ and $\boldsymbol{\mathfrak{D}}^{+} \leftarrow \left\{\mathfrak{D}_1, \ldots, \mathfrak{D}_{attr-1}, \mathfrak{D}_{attr}^{+}, \mathfrak{D}_{attr+1}, \ldots, \mathfrak{D}_d\right\}$

9: If $\mathfrak{D}_{attr}^{-} \neq \emptyset$, compute the left subtree recursively:
   $N_t.left \leftarrow$ MediAdaBoost$\big(\boldsymbol{X}, \boldsymbol{y}, \boldsymbol{w}_{t+1}^{-}, \boldsymbol{\mathfrak{D}}^{-}, t+1, T, F_t - \beta_t\big)$

10: If $\mathfrak{D}_{attr}^{+} \neq \emptyset$, compute the right subtree recursively:
   $N_t.right \leftarrow$ MediAdaBoost$\big(\boldsymbol{X}, \boldsymbol{y}, \boldsymbol{w}_{t+1}^{+}, \boldsymbol{\mathfrak{D}}^{+}, t+1, T, F_t + \beta_t\big)$

11: If $\mathfrak{D}_{attr}^{-} = \emptyset$, prune impossible left branch by returning $N_t.right$
   Else If $\mathfrak{D}_{attr}^{+} = \emptyset$, prune impossible right branch by returning $N_t.left$
   Else Return the subtree N_t

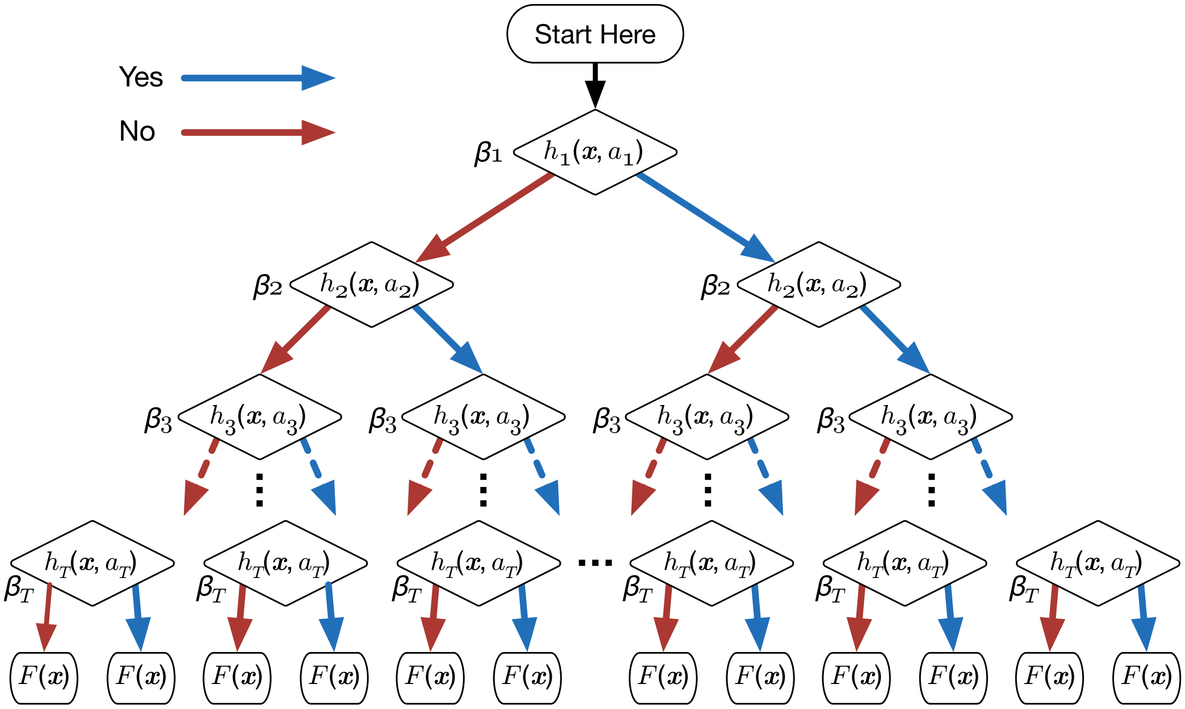

Figure S1: The model learned by AdaBoost represented as a decision tree.

membership to a branch. This acceleration term diversifies the ensembles represented by the tree and accelerates each path's convergence to a prediction at a terminal node.

First, we introduce the MediAdaBoost (MAB) algorithm as Algorithm 1, which grows a decision tree via a modified version of AdaBoost (9). At each node of the tree, MAB trains a weak learner to focus on the data instances that previous nodes have misclassified, as in AdaBoost (Algorithm 1, lines 3-6). In addition, MAB incorporates an acceleration term (second terms in lines 6a and 6b) to penalize instances whose labels disagree with the tree branch, focusing each branch more on instances that seem to have higher probability of following the corresponding path, as in fuzzy logic (15). While growing the tree, it also prunes (line 11) impossible paths based on previous decisions on the path to the root (lines 7-8).

Now we show that this algorithm is obtained if the expected value of the exponential loss function $L(F) = \mathbb{E}\big(e^{-yF(\boldsymbol{x})}\big)$ is minimized with respect to the ensemble classification rule $F(\boldsymbol{x})$ using an additive logistic regression model via Newton-like updates (10). Our argument is similar to that presented by Friedman et al. (10), but includes the acceleration term based on a membership function to diversify the ensembles and speed their convergence.

Let $L(F) = \mathbb{E}\big(e^{-yF(\boldsymbol{x})}\big)$ be the loss function of the MediBoost tree at an arbitrary terminal node N_T. Now, let us assume that we have a current estimate of the function $F_{T-1}(\boldsymbol{x})$ corresponding to a tree of depth $T-1$ and seek to improve this estimate by adding an additional split at one of the terminal nodes N_{T-1} that will define two more terminal nodes, children of N_{T-1},

using an additive step:

$$F_T(\boldsymbol{x}) = F_{T-1}(\boldsymbol{x}) + \beta_T h_T(\boldsymbol{x}, a_T) \ , \tag{2}$$

where β_T is a constant, and $h_T(\boldsymbol{x}, a_T) \in \{-1, +1\}$ represents the classification of each observation with decision predicate a_T to split the observations at N_T. The new loss function will be:

$$L(F_{T-1}(\boldsymbol{x}) + \beta_T h_T(\boldsymbol{x}, a_T)) = \mathbb{E}\big(\exp(-yF_{T-1}(\boldsymbol{x}) - y\beta_T h_T(\boldsymbol{x}, a_T))\big) \ . \tag{3}$$

Taking into account that $F_{T-1}(\boldsymbol{x})$ is fixed and expanding $\exp\left(-yF_{T-1}(\boldsymbol{x}) - y\beta_T h_T(\boldsymbol{x}, a_T)\right)$ around $h_T = h_T(\boldsymbol{x}, a_T) = 0$ (for some predicate a_T) as a second-order polynomial (for a fixed β_T and $\boldsymbol{x}$) we obtain:

$$L(F_{T-1}(\boldsymbol{x}) + \beta_T h_T) \approx \mathbb{E}\Big(e^{-yF_{T-1}(\boldsymbol{x})}\left(1 - y\beta_T h_T + \beta_T^2 y^2 h_T^2/2\right)\Big) \ . \tag{4}$$

Since $y \in \{-1, +1\}$ and $h_T \in \{-1, +1\}$, we have $y^2 = 1$ and $h_T^2 = 1$, so:

$$L(F_{T-1}(\boldsymbol{x}) + \beta_T h_T) \approx \mathbb{E}\Big(e^{-yF_{T-1}(\boldsymbol{x})}\left(1 - y\beta_T h_T + c^2/2\right)\Big) \ , \tag{5}$$

where c is a constant. Minimizing Equation 5 with respect to h_T for a fixed x yields:

$$a_T = \arg\min_a \mathbb{E}_{\mathbf{w}}\big(1 - y\beta_T h_T(x, a) + c^2/2 \mid \boldsymbol{x}\big) \ , \tag{6}$$

where $\mathbb{E}_{\mathbf{w}}(\cdot \mid \boldsymbol{x})$ refers to the weighted conditional expectation in which the weight of each instance $(\boldsymbol{x}_i, y_i)$ is given by

$$w(i) = e^{-yF_{T-1}(\boldsymbol{x}_i)} M(\boldsymbol{x}_i, T-1) \ ,$$

with a membership function or acceleration term, $M(\boldsymbol{x}, T-1)$, that emphasizes instances with predicted labels that agree with the corresponding branch of the tree. The introduction of this function is the key step that leads to MediAdaBoost, differentiates our algorithm from Discrete AdaBoost (10), and makes each path through the tree converge to a different ensemble of nodes.

Following similar steps taken by Friedman et al (10), we have that if $\beta_T > 0$, Equation 6 is equivalent to

$$\begin{aligned} a_T &= \arg\max_a \mathbb{E}_{\mathbf{w}}(yh_T(\boldsymbol{x}, a) \mid \boldsymbol{x}) \\ &= \arg\min_a -\mathbb{E}_{\mathbf{w}}(yh_T(\boldsymbol{x}, a) \mid \boldsymbol{x}) = \arg\min_a \mathbb{E}_{\mathbf{w}}(y - h_T(\boldsymbol{x}, a))^2/2 - 1 \ , \end{aligned} \tag{7}$$

where we have again taken into consideration that $y^2 = 1$ and $(h_T(\boldsymbol{x}, a))^2 = 1$.

Equation 7 indicates that in order to minimize the expected loss, $h_T(\boldsymbol{x}, a_T)$ can be obtained using a weighted least square minimization over the training data. Given $h_T(\boldsymbol{x}, a_T)$, we can obtain β_T as:

$$\beta_T = \arg\min_\beta \mathbb{E}_{\mathbf{w}}\big(\exp\left(-\beta y h_T(\boldsymbol{x}, a_T)\right)\big) \ , \tag{8}$$

which can be shown to be:

$$\beta_T = \frac{1}{2} \log \left(\frac{1 - err_T}{err_T} \right) , \tag{9}$$

where $err_T = \mathbb{E}_{\mathbf{w}}\big(\mathbb{1}(y_i \neq h_T(\boldsymbol{x}_i, a_T))\big)$ with $\mathbb{1}(p) = \begin{cases} 1 & \text{if predicate } p \text{ is true} \\ 0 & \text{otherwise} \end{cases}$.

Therefore, the new function at N_T is given by $F_T(\boldsymbol{x}) = F_{T-1}(\boldsymbol{x}) + \frac{1}{2} \log \left(\frac{1-err_T}{err_T} \right) h_T(\boldsymbol{x}, a_T)$, where $h_T(\boldsymbol{x}, a_T)$ is the decision stump that results from solving Equation 7. Let $\{N_1, \ldots, N_T\}$ denote the path from the root node to N_T. To yield MAB, we set the acceleration term to be:

$$M(\boldsymbol{x}, T-1) = \exp\left(-A \sum_{t=1}^{T-1} \mathbb{1}\Big(h_t(\boldsymbol{x}, a_t) = -child(N_t, N_{t+1}) \Big) \right) , \tag{10}$$

where A is an acceleration constant and $child(N_t, N_{t+1}) = \begin{cases} -1 & \text{if } N_t.left = N_{t+1} \\ +1 & \text{if } N_t.right = N_{t+1} \end{cases}$, thereby penalizing the weight of $\boldsymbol{x}$ by e^{-A} each time the instance is predicted to belong to a different path. If A is set to 0, then every path through the resulting tree is identical to the AdaBoost ensemble for the given problem. As the constant A increases, the resulting MAB tree converges faster and the paths through the tree represent increasingly diverse ensembles. MAB also prunes branches that are impossible to reach by tracking the valid domain for every attribute and eliminating impossible-to-follow paths during the training process. As a final step, we can post-prune the tree bottom-up by recursively eliminating the parent nodes of leaves with identical predictions, further compacting the MediBoost tree.

S4 Generalization of MediBoost's Loss Function via Gradient Boosting

In this section, we generalize the MediBoost framework to any loss function using the gradient boosting framework. As in the case of MAB, we assume that we have a current estimate of the function $F_{T-1}(\boldsymbol{x})$ corresponding to a tree of depth $T-1$ and seek to improve this estimate by adding an additional split at one of the terminal nodes N_{T-1} that will define two more terminal nodes, children of N_{T-1}, using an additive step. The function at depth T is then given by $F_T(\boldsymbol{x}) = F_{T-1}(\boldsymbol{x}) + \rho_T h'_T(\boldsymbol{x}, a_T, \boldsymbol{b}_T)$, where $\rho_T \in \mathbb{R}$ and the weak learner h'_T is given by:

$$\begin{aligned} h'_T(\boldsymbol{x}, a_T, \boldsymbol{b}_T) &= b_T^- \mathbb{1}(\boldsymbol{x} \in R_-) + b_T^+ \mathbb{1}(\boldsymbol{x} \in R_+) \\ &= \sum_{j \in \{-,+\}} b_T^j \mathbb{1}(\boldsymbol{x} \in R_j) , \end{aligned} \tag{11}$$

where $\boldsymbol{b}_T = [b_T^-, b_T^+] \in \mathbb{R}^2$, and R_- and R_+ are disjoint partitions of $\mathcal{X}$ defined by the predicate a_T. We can define a loss function over one observation $(\boldsymbol{x}_i, y_i)$ as:

$$\ell(y_i, F_T(\boldsymbol{x}_i)) = \ell\big(y_i, F_{T-1}(\boldsymbol{x}_i) + \rho_T h'_T(\boldsymbol{x}_i, a_T, \boldsymbol{b}_T)\big) , \tag{12}$$

and a loss function over all observations as

$$L = \sum_{i=1}^{N} \ell\big(y_i, F_{T-1}(\boldsymbol{x}_i) + \rho_T h'_T(\boldsymbol{x}_i, a_T, \boldsymbol{b}_T)\big) M(\boldsymbol{x}_i, T-1) \ , \tag{13}$$

where $M(\boldsymbol{x}_i, T-1)$ is a membership function of the observation $\boldsymbol{x}_i$ at node N_{T-1}, as defined in the previous section. We are interested in finding $\{\rho_T, a_T, \boldsymbol{b}_T\}$ that minimize Equation 13, which can be interpreted as the expected value of the loss function over a discrete number of observations.

Now, using a greedy stage-wise approach to minimize Equation 13 and following Friedman's gradient boosting formulation (11), $\rho_T h'_T(\boldsymbol{x}_i, a_T, \boldsymbol{b}_T)$ can be interpreted as the best greedy step to minimize Equation 13. The step direction is the decision stump $h'_T(\boldsymbol{x}_i, a_T, \boldsymbol{b}_T)$, parameterized by $\{a_T, \boldsymbol{b}_T\}$, and ρ_T is the coefficient. We, therefore, seek to find $\rho_T h'_T(\boldsymbol{x}_i, a_T, \boldsymbol{b}_T)$ that is closest to the gradient $\frac{\partial \ell(y_i, F_{T-1}(\boldsymbol{x}_i))}{\partial F_{T-1}(\boldsymbol{x}_i)}$. According to Friedman (11), one solution is to find $\{\rho_T, a_T, \boldsymbol{b}_T\}$ that minimizes the quadratic loss to approximating the "pseudo-responses" $\left\{\tilde{y}_i = -\frac{\partial \ell(y_i, F_{T-1}(\boldsymbol{x}_i))}{\partial F_{T-1}(\boldsymbol{x}_i)}\right\}_{i=1}^{N}$; in our case, this can be accomplished by solving

$$\{\rho_T, a_T, \boldsymbol{b}_T\} = \arg\min_{\rho, a, \boldsymbol{b}} \sum_{i=1}^{N} \left[-\frac{\partial \ell(y_i, F_{T-1}(\boldsymbol{x}_i))}{\partial F_{T-1}(\boldsymbol{x}_i)} - \rho h'_T(\boldsymbol{x}_i, a, \boldsymbol{b}) \right]^2 M(\boldsymbol{x}_i, T-1) \ . \tag{14}$$

To approximate the solution of Equation 14 efficiently (11), we first find the predicate a_T by fitting the decision stump to the training data using the pseudo-responses as the labels: $\{\boldsymbol{x}_i, \tilde{y}_i\}_{i=1}^{N}$. Once a_T has been found, we can use the quadratic Taylor expansion of Equation 12:

$$\begin{aligned} \ell(y_i, F_T(\boldsymbol{x}_i)) = \ell(y_i, F_{T-1}(\boldsymbol{x}_i)) &+ \frac{\partial \ell(y_i, F_{T-1}(\boldsymbol{x}_i))}{\partial F_{T-1}(\boldsymbol{x}_i)} \rho_T h'_T(\boldsymbol{x}_i, a_T, \boldsymbol{b}_T) \\ &+ \frac{1}{2} \frac{\partial^2 \ell(y_i, F_{T-1}(\boldsymbol{x}_i))}{\partial^2 F_{T-1}(\boldsymbol{x}_i)} (\rho_T h'_T(\boldsymbol{x}_i, a_T, \boldsymbol{b}_T))^2 \end{aligned} \tag{15}$$

in combination with Equation 13 to obtain the values of ρ_T and $\boldsymbol{b}_T$. For notational convenience, let the first and second derivatives of the loss function be denoted by $g_i = \frac{\partial \ell(y_i, F_{T-1}(\boldsymbol{x}_i))}{\partial F_{T-1}(\boldsymbol{x}_i)}$ and $k_i = \frac{\partial^2 \ell(y_i, F_{T-1}(\boldsymbol{x}_i))}{\partial^2 F_{T-1}(\boldsymbol{x}_i)}$. Equation 13 can then be rewritten as:

$$L = \sum_{i=1}^{N} \left[\ell(y_i, F_{T-1}(\boldsymbol{x}_i)) + g_i \rho_T h'_T(\boldsymbol{x}_i, a_T, \boldsymbol{b}_T) + \frac{k_i}{2} (\rho_T h'_T(\boldsymbol{x}_i, a_T, \boldsymbol{b}_T))^2 \right] M(\boldsymbol{x}_i, T-1) \ . \tag{16}$$

Finally, let us realize that:

$$\rho_T h'_T(\boldsymbol{x}_i, a_T, \boldsymbol{b}_T) = \sum_{j \in \{-,+\}} c_T^j \mathbb{1}(\boldsymbol{x}_i \in R_j) \ , \tag{17}$$

where $c_T^- = \rho_T b_T^-$ and $c_T^+ = \rho_T b_T^+$. Substituting Equation 17 into Equation 16, we have:

$$L = \sum_{i=1}^{N} \Bigg[\ell(y_i, F_{T-1}(\boldsymbol{x}_i)) + g_i \sum_{j \in \{-,+\}} c_T^j \mathbb{1}(\boldsymbol{x}_i \in R_j) + \frac{1}{2} k_i \Bigg(\sum_{j \in \{-,+\}} c_T^j \mathbb{1}(\boldsymbol{x}_i \in R_j) \Bigg)^2 \Bigg] M(\boldsymbol{x}_i, T-1) \ . \tag{18}$$

We are interested in finding the c_T^- and c_T^+ that minimize Equation 18 given the predicate a_T we fit to the data labeled with the pseudo-responses. We can, therefore, drop out terms from the objective function that do not depend on c_T^- and c_T^+, and rearrange to obtain:

$$L = \sum_{j \in \{-,+\}} \Bigg(c_T^j \sum_{\boldsymbol{x}_i \in \boldsymbol{X}_j} g_i M(\boldsymbol{x}_i, T-1) + \frac{1}{2} \left(c_T^j\right)^2 \sum_{\boldsymbol{x}_i \in \boldsymbol{X}_j} k_i M(\boldsymbol{x}_i, T-1) \Bigg) \ , \tag{19}$$

where $\boldsymbol{X}_j = \boldsymbol{X} \bigcap R_j$ represents the observations that belong to R_j. Finally, we can find c_T^- and c_T^+ by solving:

$$\{c_T^-, c_T^+\} = \arg\min_{c_T^-, c_T^+} \sum_{j \in \{-,+\}} \left[G_T^j c_T^j + \frac{1}{2} K_T^j \left(c_T^j\right)^2 \right] \ , \tag{20}$$

where we have defined

$$G_T^j = \sum_{\boldsymbol{x}_i \in \boldsymbol{X}_j} g_i M(\boldsymbol{x}_i, T-1) \qquad K_T^j = \sum_{\boldsymbol{x}_i \in \boldsymbol{X}_j} k_i M(\boldsymbol{x}_i, T-1) \ .$$

The solution to Equation 20 is then given by:

$$c_T^- = -\frac{G_T^-}{K_T^-} \qquad c_T^+ = -\frac{G_T^+}{K_T^+} \ , \tag{21}$$

finishing the derivation. The complete general MediBoost framework is given as Algorithm 2.

S5 Regularization and Shrinkage in MediBoost

The formulation of the MediBoost framework for general loss functions also makes MediBoost suitable for regularization—a task that is more difficult on CART or ID3. In fact in those cases, regularization is usually limited to controlling the depth of the tree.

To incorporate regularization into the MediBoost framework as presented in Section S4, we can simply add an L2-norm penalization on c_T^- and c_T^+ to Equation 20:

$$\{c_T^-, c_T^+\} = \arg\min_{c_T^-, c_T^+} \sum_{j \in \{-,+\}} \left[G_T^j c_T^j + \frac{1}{2} K_T^j \left(c_T^j\right)^2 + \lambda \left(c_T^j\right)^2 \right] \ , \tag{22}$$

Algorithm 2 MediBoost$(\boldsymbol{X}, \boldsymbol{y}, \boldsymbol{w}_t, LR, \lambda, \boldsymbol{\mathfrak{D}}, t, T, F_{t-1}, C_t)$

Inputs:

- training data $\boldsymbol{X} = \{\boldsymbol{x}_1, \ldots, \boldsymbol{x}_n\}$ with corresponding labels $\boldsymbol{y} = \{y_1, \ldots, y_n\}$, where $\boldsymbol{x}_i \in \mathcal{X}$ for some d-dimensional feature space $\mathcal{X}$, and $y_i \in \{-1, +1\}$
- instance weights $\boldsymbol{w}_t \in \mathbb{R}^n$, which defaults to a uniform distribution initially
- learning rate $LR \in (0, 1]$, and regularization constant $\lambda \in [0, +\infty]$
- valid domain of each attribute $\boldsymbol{\mathfrak{D}} = \{\mathfrak{D}_1, \ldots, \mathfrak{D}_d\}$ (for continuous attributes, $\mathfrak{D}_i$ is a valid interval; for discrete or categorical attributes, $\mathfrak{D}_i$ is a set of valid values)
- node index t, which defaults to 1, and the total number of boosting iterations T
- prediction function $F_{t-1}(\boldsymbol{x}) \in \mathbb{R}^N$, which defaults initially to $F_0(\boldsymbol{x}) = \frac{1}{2} \log \frac{1+\text{mean}(\boldsymbol{y})}{1-\text{mean}(\boldsymbol{y})}$
- accumulated sum of coefficients C_t, which defaults to 0

Outputs: the root node of a decision tree

1: Create a root node N_t for the subtree

2: If $t > T$, Return the single-node subtree N_t with label sign(C_t)

3: Fit weak classifier $h'_t(\boldsymbol{x}, a_t, \boldsymbol{b}_t) : \mathcal{X} \mapsto \mathbb{R}$ by finding the stump with decision rule a_t on attribute $attr$ that minimizes the weighted least square error on approximating the pseudo-responses $-\frac{\partial \ell(y, F_{t-1}(\boldsymbol{x}))}{\partial F_{t-1}(\boldsymbol{x})}$ over $\boldsymbol{X}$ with observation weights $\boldsymbol{w}_t$ by solving

$$a_t \leftarrow \arg\min_a \sum_{i=1}^{N} w_t(i) \left[-\frac{\partial \ell\left(y_i, F_{t-1}(\boldsymbol{x}_i)\right)}{\partial F_{t-1}(\boldsymbol{x}_i)} - h'_t(\boldsymbol{x}_i, a)) \right]^2$$

4: Update the node weights for the left (-) and right (+) subtrees. For $j \in \{-, +\}$, do:

$$c_t^j \leftarrow - \frac{LR \times \sum_{\boldsymbol{x}_i \in \boldsymbol{X}_j} \frac{\partial \ell(y_i, F_{t-1}(\boldsymbol{x}_i))}{\partial F_{t-1}(\boldsymbol{x}_i)} M(\boldsymbol{x}_i, t-1)}{\lambda + \sum_{\boldsymbol{x}_i \in \boldsymbol{X}_j} \frac{\partial^2 \ell(y_i, F_{t-1}(\boldsymbol{x}_i))}{\partial^2 F_{t-1}(\boldsymbol{x}_i)} M(\boldsymbol{x}_i, t-1)}$$

5: Let $\mathfrak{D}(a_t)$ represent the domain of attribute $attr$ when a_t is true, and $\mathfrak{D}^c(a_t)$ its complement

6: Compute valid domains for all attributes for the left (-) and right (+) subtrees:

a. Let $\mathfrak{D}_{attr}^- \leftarrow \mathfrak{D}_{attr} \bigcap \mathfrak{D}^c(a_t)$ and $\boldsymbol{\mathfrak{D}}^- \leftarrow \left\{\mathfrak{D}_1, \ldots, \mathfrak{D}_{attr-1}, \mathfrak{D}_{attr}^-, \mathfrak{D}_{attr+1}, \ldots, \mathfrak{D}_d\right\}$

b. Let $\mathfrak{D}_{attr}^+ \leftarrow \mathfrak{D}_{attr} \bigcap \mathfrak{D}(a_t)$ and $\boldsymbol{\mathfrak{D}}^+ \leftarrow \left\{\mathfrak{D}_1, \ldots, \mathfrak{D}_{attr-1}, \mathfrak{D}_{attr}^+, \mathfrak{D}_{attr+1}, \ldots, \mathfrak{D}_d\right\}$

7: Update the instance weights for the left (-) and right(+) subtrees:

$\forall \boldsymbol{x}_i \in \boldsymbol{X}$, do: $\quad w_{t+1}^-(i) \leftarrow M(\boldsymbol{x}_i, t) \in \mathbb{R} \quad$ and $\quad w_{t+1}^+(i) \leftarrow M(\boldsymbol{x}_i, t) \in \mathbb{R}$

8: $F_t(\boldsymbol{x}_i) \leftarrow F_{t-1}(\boldsymbol{x}_i) + c_t^- \mathbb{1}(\neg a_t(\boldsymbol{x}_i)) + c_t^+ \mathbb{1}(a_t(\boldsymbol{x}_i)) \;\; \forall \boldsymbol{x}_i \in \boldsymbol{X}$

9: If $\mathfrak{D}_{attr}^- \neq \emptyset$, compute the left subtree recursively:

$N_t.left \leftarrow$ MediBoost$\left(\boldsymbol{X}, \boldsymbol{y}, LR, \lambda, \boldsymbol{w}_{t+1}^-, \boldsymbol{\mathfrak{D}}^-, t+1, T, F_t, C_t + c_t^-\right)$

10: If $\mathfrak{D}_{attr}^+ \neq \emptyset$, compute the right subtree recursively:

$N_t.right \leftarrow$ MediBoost$\left(\boldsymbol{X}, \boldsymbol{y}, LR, \lambda, \boldsymbol{w}_{t+1}^+, \boldsymbol{\mathfrak{D}}^+, t+1, T, F_t, C_t + c_t^+\right)$

11: If $\mathfrak{D}_{attr}^- = \emptyset$, prune impossible left branch by returning $N_t.right$

Else If $\mathfrak{D}_{attr}^+ = \emptyset$, prune impossible right branch by returning $N_t.left$

Else Return the subtree N_t

with the subsequent coefficients given by:

$$c_T^- = -\frac{G_T^-}{K_T^- + \lambda} \qquad\qquad c_T^+ = -\frac{G_T^+}{K_T^+ + \lambda} \ ,$$

where the regularization parameter $\lambda \in [0, \infty]$. Setting $\lambda = 0$ eliminates regularization.

The concept of shrinkage or a learning rate, which is regularly used in gradient boosting, can also be applied to MediBoost. In this case, the solutions to Equation 22 will be given by:

$$c_T^- = -\frac{LR \times G_T^-}{K_T^- + \lambda} \qquad\qquad c_T^+ = -\frac{LR \times G_T^+}{K_T^+ + \lambda} \ , \tag{23}$$

where $0 < LR \leq 1$ is the shrinkage or learning rate constant, typically assumed to be 0.1 (11,14). Each of these regularization methods can be used independently of each other. In the gradient boosting community, the use of shrinkage is the most popular and it has been interpreted as equivalent to L1-norm penalization on the weights (14).

Algorithm 2 states the general form of the MediBoost framework, incorporating the general loss function formulation from Section S4, and the regularization and shrinkage techniques discussed above. The general MediBoost framework also eliminates impossible paths through the tree during the learning process by tracking the valid domain of each attribute, and support post-pruning to eliminate unnecessary subtrees that always yield the same prediction, as described in Section S3. Using this general framework, we can create various MediBoost algorithms by choosing specific loss functions for the boosting process, as shown in the next section.

S6 LikelihoodMediBoost (LMB)

In this section, we explore a concrete instantiation of the general MediBoost framework, using negative binomial log-likelihood as the loss function and a similar membership function as the one used in MAB. Gradient boosting with binomial log-likelihood typically outperforms AdaBoost (11), and results in a more accurate algorithm with fewer ensemble members. The negative binomial log-likelihood loss function is given by.

$$\ell(y_i, F(\boldsymbol{x}_i)) = \log\left(1 + \exp(-2y_i F(\boldsymbol{x}_i))\right) \ .$$

Using the log-likelihood loss, $F(\boldsymbol{x}_i)$ can also be interpreted as one-half of the log odds ratio:

$$F(\boldsymbol{x}_i) = \frac{1}{2}\log\left(\frac{\Pr(y = 1 \mid \boldsymbol{x}_i)}{\Pr(y = -1 \mid \boldsymbol{x}_i)}\right) \ , \tag{24}$$

which justifies classifying $\boldsymbol{x}_i$ as $\operatorname{sign}(F(\boldsymbol{x}_i))$.

Algorithm 3 LikelihoodMediBoost($\boldsymbol{X}, \boldsymbol{y}, \boldsymbol{w}_t, LR, \lambda, \boldsymbol{\mathfrak{D}}, t, T, F_{t-1}, C_t, M_{t-1}$)

Inputs:

- training data $\boldsymbol{X} = \{\boldsymbol{x}_1, \ldots, \boldsymbol{x}_n\}$ with corresponding labels $\boldsymbol{y} = \{y_1, \ldots, y_n\}$, where $\boldsymbol{x}_i \in \mathcal{X}$ for some d-dimensional feature space $\mathcal{X}$, and $y_i \in \{-1, +1\}$
- instance weights $\boldsymbol{w}_t \in \mathbb{R}^n$, which defaults to a uniform distribution initially
- learning rate $LR \in (0, 1]$, and regularization constant $\lambda \in (0, +\infty)$
- valid domain of each attribute $\boldsymbol{\mathfrak{D}} = \{\mathfrak{D}_1, \ldots, \mathfrak{D}_d\}$ (for continuous attributes, $\mathfrak{D}_i$ is a valid interval; for discrete or categorical attributes, $\mathfrak{D}_i$ is a set of valid values)
- node index t, which defaults to 1, and the total number of boosting iterations T
- prediction function $F_{t-1}(\boldsymbol{x}) \in \mathbb{R}^N$, which defaults initially to $F_0(\boldsymbol{x}) = \frac{1}{2} \log \frac{1+\text{mean}(\boldsymbol{y})}{1-\text{mean}(\boldsymbol{y})}$
- accumulated sum of coefficients C_t, which defaults to 0
- cumulative acceleration term $M_{t-1}(\boldsymbol{x}) \in \mathbb{R}^N$, which defaults initially to $M_0(\boldsymbol{x}) = 1$

Output: the root node of a decision tree

1: Create a root node N_t for the subtree

2: If $t > T$, Return the single-node subtree N_t with label sign(C_t)

3: Fit weak classifier $h'_t(\boldsymbol{x}, a_t) : \mathcal{X} \mapsto \{-1, +1\}$ by finding the stump with decision rule a_t on attribute $attr$ that minimizes the weighted least square error on approximating the pseudo-responses $-\frac{\partial \ell(y, F_{t-1}(\boldsymbol{x}))}{\partial F_{t-1}(\boldsymbol{x})}$ over $\boldsymbol{X}$ with observation weights $\boldsymbol{w}_t$ by solving

$$a_t \leftarrow \arg\min_a \sum_{i=1}^{N} w_t(i) \left[\frac{-2y_i}{(1 + e^{2y_i F_{t-1}(\boldsymbol{x}_i)})} - h'_t(\boldsymbol{x}_i, a) \right]^2$$

4: Update the node weights for the left (-) and right (+) subtrees. For $j \in \{-, +\}$, do:

$$c_t^j \leftarrow -\frac{LR \times \sum_{\boldsymbol{x}_i \in \boldsymbol{X}_j} g_i M_{t-1}(\boldsymbol{x}_i)}{\lambda + \sum_{\boldsymbol{x}_i \in \boldsymbol{X}_j} \|g_i\| \left(2 - \|g_i\|\right) M_{t-1}(\boldsymbol{x}_i)} \quad \text{where } g_i = \frac{-2y_i}{(1+\exp(2y_i F_{t-1}(\boldsymbol{x}_i)))}$$

5: Let $\mathfrak{D}(a_t)$ represent the domain of attribute $attr$ when a_t is true, and $\mathfrak{D}^c(a_t)$ its complement

6: Compute valid domains for all attributes for the left (-) and right (+) subtrees:

a. Let $\mathfrak{D}^-_{attr} \leftarrow \mathfrak{D}_{attr} \bigcap \mathfrak{D}^c(a_t)$ and $\boldsymbol{\mathfrak{D}}^- \leftarrow \left\{\mathfrak{D}_1, \ldots, \mathfrak{D}_{attr-1}, \mathfrak{D}^-_{attr}, \mathfrak{D}_{attr+1}, \ldots, \mathfrak{D}_d\right\}$

b. Let $\mathfrak{D}^+_{attr} \leftarrow \mathfrak{D}_{attr} \bigcap \mathfrak{D}(a_t)$ and $\boldsymbol{\mathfrak{D}}^+ \leftarrow \left\{\mathfrak{D}_1, \ldots, \mathfrak{D}_{attr-1}, \mathfrak{D}^+_{attr}, \mathfrak{D}_{attr+1}, \ldots, \mathfrak{D}_d\right\}$

7: Update the instance weights for the left (-) and right (+) subtrees:

a. $w^-_{t+1}(i) \leftarrow \frac{w_t(i)}{Z^-_t} \frac{\exp(-A\mathbb{1}(a_t(\boldsymbol{x}_i)))}{\exp(-A\mathbb{1}(\neg a_t(\boldsymbol{x}_i)))+\exp(-A\mathbb{1}(a_t(\boldsymbol{x}_i)))} \quad \forall \boldsymbol{x}_i \in \boldsymbol{X}$

b. $w^+_{t+1}(i) \leftarrow \frac{w_t(i)}{Z^+_t} \frac{\exp(-A\mathbb{1}(\neg a_t(\boldsymbol{x}_i)))}{\exp(-A\mathbb{1}(\neg a_t(\boldsymbol{x}_i)))+\exp(-A\mathbb{1}(a_t(\boldsymbol{x}_i)))} \quad \forall \boldsymbol{x}_i \in \boldsymbol{X}$

where Z^-_t and Z^+_t are normalization factors to make $\boldsymbol{w}^-_{t+1}$ and $\boldsymbol{w}^+_{t+1}$ distributions.

8: $F_t(\boldsymbol{x}_i) \leftarrow F_{t-1}(\boldsymbol{x}_i) + c^-_t \mathbb{1}(\neg a_t(\boldsymbol{x}_i)) + c^+_t \mathbb{1}(a_t(\boldsymbol{x}_i)) \;\; \forall \boldsymbol{x}_i \in \boldsymbol{X}$

9: $M^-_t(\boldsymbol{x}_i) \leftarrow M_{t-1} \exp(-A\mathbb{1}(a_t(\boldsymbol{x}_i)))$ and $M^+_t(\boldsymbol{x}_i) \leftarrow M_{t-1} \exp(-A\mathbb{1}(\neg a_t(\boldsymbol{x}_i))) \;\; \forall \boldsymbol{x}_i \in \boldsymbol{X}$

10: If $\mathfrak{D}^-_{attr} \neq \emptyset$, compute the left subtree recursively:
$N_t.left \leftarrow$ LikelihoodMediBoost$\left(\boldsymbol{X}, \boldsymbol{y}, LR, \lambda, \boldsymbol{w}^-_{t+1}, \boldsymbol{\mathfrak{D}}^-, t+1, T, F_t, C_t + c^-_t, M^-_t\right)$

11: If $\mathfrak{D}^+_{attr} \neq \emptyset$, compute the right subtree recursively:
$N_t.right \leftarrow$ LikelihoodMediBoost$\left(\boldsymbol{X}, \boldsymbol{y}, LR, \lambda, \boldsymbol{w}^+_{t+1}, \boldsymbol{\mathfrak{D}}^+, t+1, T, F_t, C_t + c^+_t, M^+_t\right)$

12: If $\mathfrak{D}^-_{attr} = \emptyset$, prune impossible left branch by returning $N_t.right$
Else If $\mathfrak{D}^+_{attr} = \emptyset$, prune impossible right branch by returning $N_t.left$
Else Return the subtree N_t

To incorporate this loss function into the MediBoost framework, we first take the first and second derivative of the loss function:

$$g_i = \frac{\partial \ell(y_i, F(\boldsymbol{x}_i))}{\partial F(\boldsymbol{x}_i)} = \frac{-2y_i}{(1 + \exp(2y_i F(\boldsymbol{x}_i)))} \tag{25}$$

$$k_i = \frac{\partial^2 \ell(y_i, F(\boldsymbol{x}_i))}{\partial^2 F(\boldsymbol{x}_i)} = \|g_i\| \left(2 - \|g_i\|\right) \quad . \tag{26}$$

Using these derivatives, we can compute G_T^-, G_T^+, K_T^-, and K_T^-, and then combine these with Equation 23 to compute the coefficients at each split as:

$$c_T^- = -\frac{LR \times G_T^-}{K_T^- + \lambda} \qquad\qquad c_T^+ = -\frac{LR \times G_T^+}{K_T^+ + \lambda} \quad .$$

Incorporating these derivatives and coefficients into the general MediBoost framework (Algorithm 2) yields the LikelihoodMediBoost (LMB) algorithm (Algorithm 3).

S7 Evaluation Methodology

Our evaluation compared the MAB and LMB MediBoost algorithms to standard decision tree induction (ID3, CART) and ensemble methods (LogitBoost and Random Forests) on 13 data sets, corresponding to all binary classification problems in the field of Life Sciences within the UCI Repository (Table S1). For each data set, any missing values were imputed with either the mean or the mode of the corresponding feature, depending on whether the features were continuous or categorical. We added additional binary features (one per each original feature) to encode whether or not the corresponding value was missing.

Results were averaged over 5 trials of 5-fold cross-validation on each data set, recording the error on the held-out test fold. Each algorithm has a number of hyperparameters, which were tuned via an additional 5-fold cross-validation on the training data in each case; then, the model was constructed using all available training folds and evaluated on the test fold. The hyperparameters adjusted for each algorithm are:

MediBoost (MAD and LMB): tree depth and acceleration parameter

ID3: tree depth

CART: tree depth

LogitBoost: Number of stump trees on the ensemble

Random Forests: Number of variables selected in each random sub-sampling.

In addition, LogitBoost used decision stumps as the weak learners with a learning rate of 0.1, and Random Forests used 300 decision trees in the ensemble. The MediBoost algorithms were run with learning rates of $LR \in \{0.1, 1.0\}$ and $\lambda = 0$.

Table S1: Life science data sets used in the evaluation.

**Data Set Name**	**Link**
Acute Inflamation (Bladder)	https://archive.ics.uci.edu/ml/datasets/Acute+Inflammations<br>Instances = 120 ; Attributes = 6 ; Missing Values = No
Acute Inflamation (Nephritis)	https://archive.ics.uci.edu/ml/datasets/Acute+Inflammations<br>Instances = 120 ; Attributes = 6 ; Missing Values = No
Arrhythmia	https://archive.ics.uci.edu/ml/datasets/Arrhythmia<br>Instances = 472 ; Attributes = 279 ; Missing Values = Yes
Breast Cancer Wisconsin (Original)	https://archive.ics.uci.edu/ml/datasets/Breast+Cancer+Wisconsin+(Original)<br>Instances = 699 ; Attributes = 10 ; Missing Values = Yes
Diabetic Retinopathy Debrecen	https://archive.ics.uci.edu/ml/datasets/Diabetic+Retinopathy+Debrecen+Data+Set<br>Instances = 1151 ; Attributes = 20 ; Missing Values = No
Fertility	https://archive.ics.uci.edu/ml/datasets/Fertility<br>Instances = 100 ; Attributes = 10 ; Missing Values = No
Hepatitis	https://archive.ics.uci.edu/ml/datasets/Hepatitis<br>Instances = 155 ; Attributes = 19 ; Missing Values = Yes
Mammographic Mass	https://archive.ics.uci.edu/ml/datasets/Mammographic+Mass<br>Instances = 961 ; Attributes = 6 ; Missing Values = Yes
Parkinson	https://archive.ics.uci.edu/ml/datasets/Parkinsons<br>Instances = 197 ; Attributes = 23 ; Missing Values = No
Pima Indians diabetes	https://archive.ics.uci.edu/ml/datasets/Pima+Indians+Diabetes<br>Instances = 768 ; Attributes = 8 ; Missing Values = Yes
Heart Disease (Cleveland)	https://archive.ics.uci.edu/ml/datasets/Heart+Disease<br>Instances = 303 ; Attributes = 75 ; Missing Values = Yes
SPECT Heart	https://archive.ics.uci.edu/ml/datasets/SPECT+Heart<br>Instances = 267 ; Attributes = 22 ; Missing Values = No
Thoracic Surgery	https://archive.ics.uci.edu/ml/datasets/Thoracic+Surgery+Data<br>Instances = 470 ; Attributes = 17 ; Missing Values = No

S8 Additional Results

This section presents additional results beyond those in the main paper.

Table S2 presents a detailed comparison of LMB with two learning rates against ID3, CART, LogitBoost, and Random Forests on the different medical problems. We report the mean and standard deviation of the test error on held-out data, reweighting the error to emphasize all classes equally and averaging it over 5 trials of 5-fold cross-validation. This comparison is also represented visually in Fig. S2. Table S3 summarizes these results for LMB with $LR = 0.1$, showing the total numbers of wins and losses against the other algorithms. (A similar table is presented in the main article for LMB with $LR = 1.0$; both cases show comparable results.)

We also conducted a similar comparison of MAB with the decision tree algorithms (ID3 and

Table S2: LMB vs different algorithms. This table compares the equal-class-weighted error (averaged over 5 trials of 5-fold cross-validation) for different medical problems and algorithms. For each problem, mean ± standard deviation are shown.

**Data Sets**	**LMB** ($LR = 0.01$)	**LMB** ($LR = 1.0$)	**ID3**	**CART**	**LogitBoost**	**Random Forests**
Acute Inflamation (Bladder)	0 ±0	0 ±0	0.005 ±0.011	0.012 ±0.010	0 ±0	0 ±0
Acute Inflamation (Nephritis)	0.004 ±0.009	0.004 ±0.009	0.008 ±0.011	0.004 ±0.009	0.004 ±0.009	0.005 ±0.009
Arrhythmia	0.219 ±0.009	0.216 ±0.023	0.261 ±0.006	0.272 ±0.017	0.187 ±0.009	0.205 ±0.011
Breast Cancer Wisconsin (Original)	0.038 ±0.004	0.045 ±0.005	0.048 ±0.005	0.048 ±0.002	0.046 ±0.002	0.025 ±0.003
Diabetic Retinopathy Debrecen	0.343 ±0.005	0.330 ±0.006	0.363 ±0.011	0.358 ±0.005	0.337 ±0.008	0.297 ±0.005
Fertility	0.378 ±0.033	0.412 ±0.060	0.338 ±0.040	0.366 ±0.037	0.405 ±0.035	0.444 ±0.031
Hepatitis	0.398 ±0.047	0.419 ±0.022	0.514 ±0.013	0.400 ±0.039	0.374 ±0.025	0.215 ±0.038
Mammographic Mass	0.177 ±0.004	0.174 ±0.005	0.190 ±0.001	0.178 ±0.006	0.174 ±0.003	0.176 ±0.005
Parkinson	0.194 ±0.014	0.160 ±0.029	0.211 ±0.042	0.196 ±0.024	0.194 ±0.027	0.124 ±0.019
Pima Indians diabetes	0.248 ±0.017	0.268 ±0.004	0.292 ±0.014	0.275 ±0.011	0.294 ±0.011	0.253 ±0.008
Heart Disease (Cleveland)	0.184 ±0.018	0.193 ±0.019	0.389 ±0.033	0.251 ±0.020	0.170 ±0.007	0.179 ±0.009
SPECT Heart	0.278 ±0.029	0.251 ±0.022	0.299 ±0.012	0.298 ±0.025	0.301 ±0.021	0.233 ±0.027
Thoracic Surgery	0.439 ±0.030	0.416 ±0.024	0.538 ±0.063	0.465 ±0.021	0.497 ±0.009	0.440 ±0.029

CART) and ensemble methods (LogitBoost and Random Forests) on the 13 medical problems. Table S6 provides details on the average test error over all algorithms and medical problems, with the comparison shown graphically in Fig. S3.

Over most problems, these results show that LMB significantly outperforms standard decision tree algorithms (ID3 and CART) while attaining statistically indishtinguishable performance from the ensemble methods (LogitBoost and Random Forests), according to a two-tailed sign test (19). In the Fertility dataset, where LMB was outperformed by ID3 and CART, these standard decision trees also outperformed the ensemble methods.

Table S3: LMB (Learning Rate = 0.1) vs different algorithms. LMB is significantly better than ID3 and CART ($p < 0.05$), and undistinguishable from LogitBoost and Random Forests, according to a two-tailed sign test (19).

LMB vs	ID3	CART	LogitBoost	Random Forests
wins	12	11	6	4
losses	1	1	5	8
ties	0	1	2	1

Table S4: MAB vs different algorithms: comparison of equal-class-weighted error (averaged over 5 trials of 5-fold cross-validation) for different medical problems and algorithms. For each problem, mean ± standard deviation are shown.

Data Sets	MAB	ID3	CART	LogitBoost	Random Forests
Acute Inflamation (Bladder)	0 ±0	0.003 ±0.007	0.009 ±0.000	0 ±0	0 ±0
Acute Inflamation (Nephritis)	0 ±0	0 ±0	0 ±0	0 ±0	0.001 ±0.003
Arrhythmia	0.202 ±0.014	0.247 ±0.022	0.246 ±0.018	0.173 ±0.013	0.189 ±0.006
Breast Cancer Wisconsin (Original)	0.039 ±0.007	0.057 ±0.002	0.053 ±0.009	0.046 ±0.005	0.022 ±0.002
Diabetic Retinopathy Debrecen	0.342 ±0.012	0.365 ±0.009	0.365 ±0.007	0.334 ±0.004	0.307 ±0.007
Fertility	0.441 ±0.036	0.407 ±0.063	0.403 ±0.033	0.408 ±0.079	0.463 ±0.066
Hepatitis	0.405 ±0.031	0.524 ±0.007	0.375 ±0.019	0.374 ±0.028	0.211 ±0.012
Mammographic Mass	0.176 ±0.002	0.189 ±0.001	0.184 ±0.006	0.171 ±0.004	0.182 ±0.002
Parkinson	0.149 ±0.019	0.192 ±0.016	0.213 ±0.005	0.173 ±0.010	0.113 ±0.006
Pima Indians diabetes	0.261 ±0.010	0.292 ±0.010	0.273 ±0.014	0.296 ±0.009	0.250 ±0.010
Heart Disease (Cleveland)	0.190 ±0.012	0.389 ±0.019	0.253 ±0.024	0.168 ±0.005	0.187 ±0.009
SPECT Heart	0.250 ±0.005	0.284 ±0.014	0.293 ±0.028	0.291 ±0.019	0.235 ±0.023
Thoracic Surgery	0.431 ±0.032	0.513 ±0.022	0.482 ±0.015	0.508 ±0.010	0.445 ±0.011

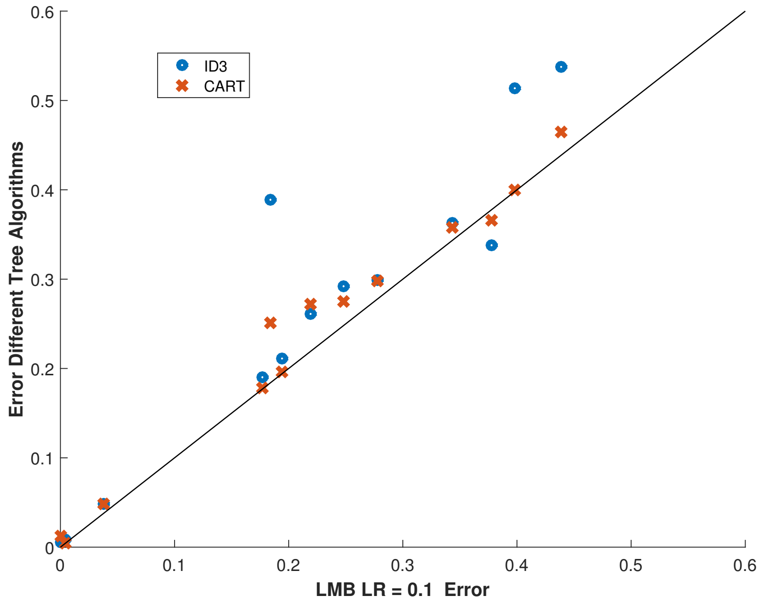

(a) LMB vs decision trees

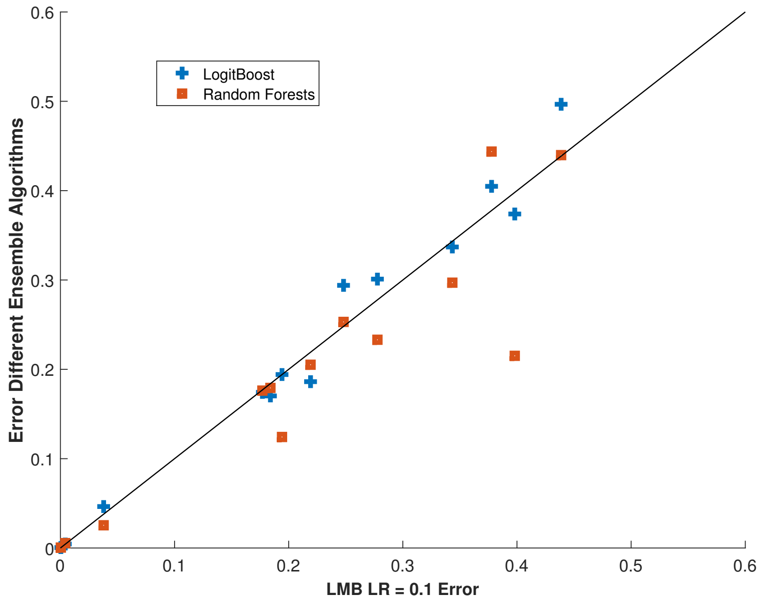

(b) LMB vs ensemble methods

Figure S2: Comparison of LMB (LR = 0.1) vs (a) ID3 and CART decision tree algorithms and (b) LogitBoost and Random Forests ensemble methods on 13 medical datasets. Points above the diagonal black line indicate results where LMB was better. LMB is significantly better than the decision tree algorithms and statistically indistinguishable from the ensemble methods.

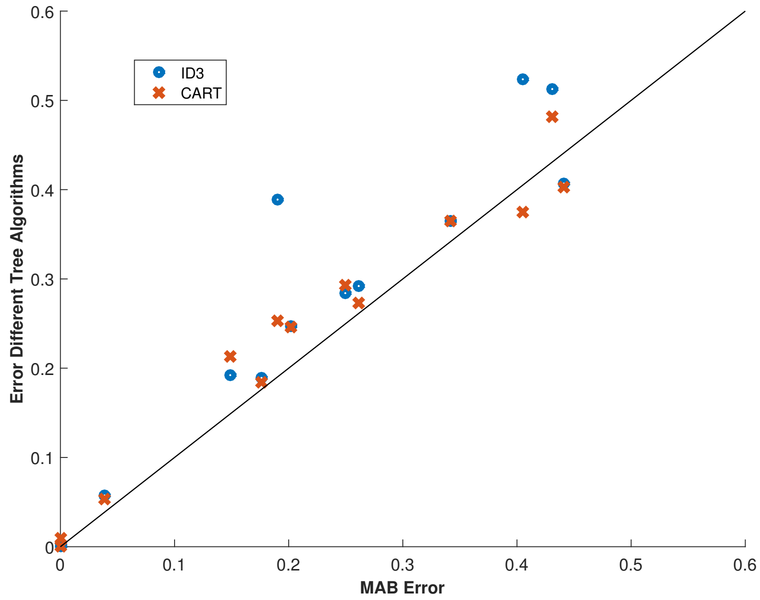

(a) MAB vs decision trees

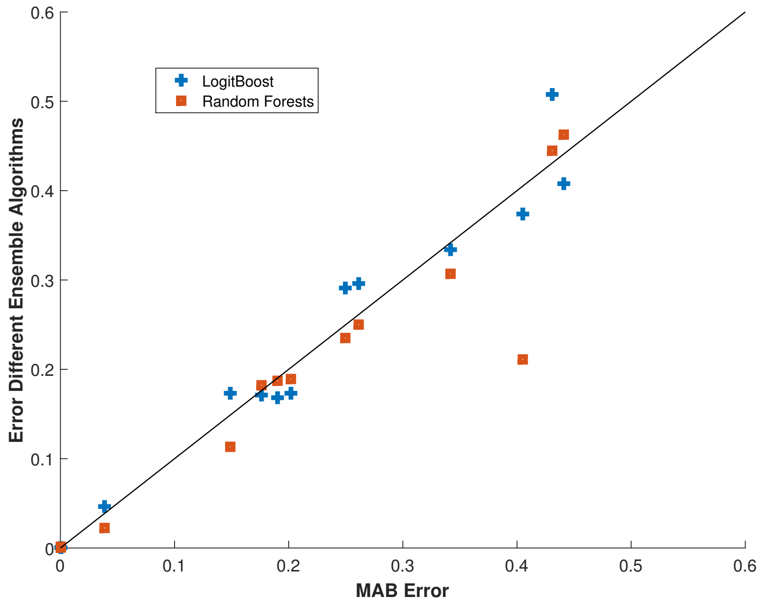

(b) MAB vs ensemble methods

Figure S3: Comparison of MAB vs (a) ID3 and CART decision tree algorithms and (b) LogitBoost and Random Forests ensemble methods on 13 medical datasets. Points above the diagonal black lines indicate experiments where MAB performed better. MAB is significantly better than the standard decision tree algorithms and indistinguishable from the ensemble methods.

Table S5: MLB vs different algorithms: comparison of AUC (averaged over 5 trials of 5-fold cross-validation) for different medical problems and algorithms. For each problem, mean ± standard deviation of AUC

Data Sets	MAB	ID3	CART	LogitBoost	Random Forests
Acute Inflamation (Bladder)	1 ±0	0.9882 ±0.0141	0.9932 ±0.0038	1 ±0.0015	0.9993 ±0
Acute Inflamation (Nephritis)	1 ±0	0.9960 ±0.0089	1±0	1±0	1 ±0
Arrhythmia	0.8547 ±0.0178	0.7247 ±0.0095	0.7463 ±0.0149	0.8913 ±0.0050	0.8788 ±0.0089
Breast Cancer Wisconsin (Original)	0.8128 ±0.0141	0.6963 ±0.0229	0.7391±0.0211	0.7221±0.0099	0.8200 ±0.0357
Diabetic Retinopathy Debrecen	0.7184 ±0.0088	0.6417 ±0.0116	0.6755 ±0.0155	0.7262 ±0.0038	0.7622 ±0.0060
Fertility	0.6070 ±0.0636	0.6242 ±0.0758	0.6094 ±0.0901	0.5747 ±0.0394	0.6120 ±0.0718
Hepatitis	0.6842 ±0.0488	0.54878 ±0.0256	0.6481 ±0.0392	0.6359±0.0125	0.8499 ±0.0339
Mammographic Mass	0.8960 ±0.0038	0.8112 ±0.0026	0.8652 ±0.0073	0.8977 ±0.0018	0.8881 ±0.0044
Parkinson	0.8962 ±0.0291	0.7718 ±0.0426	0.8113 ±0.0292	0.9231±0.0068	0.9585±0.0190
Pima Indians diabetes	0.8107 ±0.0066	0.7089 ±0.0062	0.7860 ±0.0100	0.8322 ±0.0041	0.8300 ±0.0046
Heart Disease (Cleveland)	0.8797 ±0.0133	0.6482 ±0.0092	0.8152 ±0.0223	0.8929 ±0.0072	0.8954 ±0.0056
SPECT Heart	0.8128 ±0.0141	0.6963 ±0.0229	0.7391±0.0211	0.7221±0.0099	0.8200±0.0357
Thoracic Surgery	0.6071 ±0.0157	0.5034 ±0.0108	0.5212 ±0.0209	0.5296 ±0.0165	0.6115±0.0303

Table S6: Comparison of AUC (averaged over 5 trials of 5-fold cross-validation) for randomlly permuted labels. Each For each problem, mean ± standard deviation of 100 iterations are shown.

Data Sets	MAB	ID3	CART	LogitBoost	Random Forests
Acute Inflamation (Bladder)	0.4897±0.0846	0.4950±0.0896	0.5020±0.0777	0.5065 ±0.0977	0.4986±0.0852
Acute Inflamation (Nephritis)	0.5046 ±0.1159	0.4851 ±0.1033	0.4963±0.1316	0.5033±0.1570	0.4870±0.1358
Arrhythmia	0.4939 ±0.0332	0.4970 ±0.0300	0.5047 ±0.0329	0.5020 ±0.0445	0.5058 ±0.0329
Breast Cancer Wisconsin (Original)	0.4484±0.0517	0.5048±0.0893	0.5089±0.0712	0.5767±0.0442	0.5103±0.0893
Diabetic Retinopathy Debrecen	0.5005 ±0.0203	0.4989±0.0208	0.5020±0.0270	0.4960±0.0195	0.4997±0.0270
Fertility	0.5030 ±0.0862	0.4965±0.0537	0.4959±0.0797	0.4909±0.0958	0.5002±0.0819
Hepatitis	0.3813 ±0.0485	0.5029 ±0.0115	0.5312±0.0689	0.4566±0.0845	0.5491±0.0659
Mammographic Mass	0.5000 ±0.0504	0.5017 ±0.0346	0.5043 ±0.0546	0.4982 ±0.0350	0.4993±0.0682
Parkinson	0.4753±0.0591	0.4862±0.0595	0.5202±0.0658	0.5167±0.0720	0.5195±0.0681
Pima Indians diabetes	0.4969 ±0.0326	0.5006 ±0.0326	0.5084±0.0372	0.5019 ±0.0348	0.5053 ±0.0442
Heart Disease (Cleveland)	0.4992 ±0.0490	0.4961±0.0414	0.5013±0.0569	0.5002 ±0.0524	0.5018±0.0569
SPECT Heart	0.552 ±0.0664	0.5109 ±0.0633	0.4601±0.0511	0.4877±0.06787	0.4165±0.0757
Thoracic Surgery	0.4950 ±0.0431	0.5034 ±0.0195	0.5085 ±0.0364	0.5132 ±0.00441	0.5031±0.0401
